# Facile and green synthesis of monodisperse sub-10 nm copper and tin nanoparticles using l-ascorbic acid as the reducing agent

**DOI:** 10.1039/d5ra04557f

**Published:** 2025-10-01

**Authors:** Abdennour Benabbas, Grégoire Breyton, Catherine Especel, Anthony Le Valant, Christian Ricolleau, Guillaume Wang, Tzonka Mineva, Jaysen Nelayah, Hazar Guesmi, Florence Epron

**Affiliations:** a CNRS, Université de Poitiers, Institut de Chimie des Milieux et Matériaux de Poitiers (IC2MP) Poitiers France florence.epron@univ-poitiers.fr abdennour.benabbas@univ-poitiers.fr; b Laboratoire Matériaux et Phénomènes Quantiques, Université Paris Cité – CNRS Paris France; c Institut Charles Gerhardt de Montpellier (ICGM), Université de Montpellier, CNRS, ENSCM Montpellier France

## Abstract

We first optimized a simple and low-cost polyol-based synthesis route for the preparation of stable and monodisperse sub-10 nm copper nanoparticles. Building on this robust approach, we extended the method to tin and succeeded in producing tin nanoparticles that stabilized in an unconventional α-Sn phase, which is remarkable given the metastable character of this phase under ambient conditions. The resulting α-Sn nanoparticles exhibited excellent resistance to oxidation, together with long-term colloidal stability in air, enabling further processing for potential applications. In both cases, inexpensive commercial precursors and mild conditions (80 °C, aqueous or polyol solvents, ascorbic acid as the sole reducing agent, and no inert atmosphere or additional stabilizers) were employed. The nanoparticles were characterized using TEM, UV-visible spectroscopy, ATR-FTIR, ICP-OES, and XPS.

## Abstract 2

Designing a simple and green experimental protocol for the synthesis of stable and small Cu(0) and Sn(0) nanospheres using l-ascorbic acid as the reducing agent under mild conditions.

## Graphical abstract

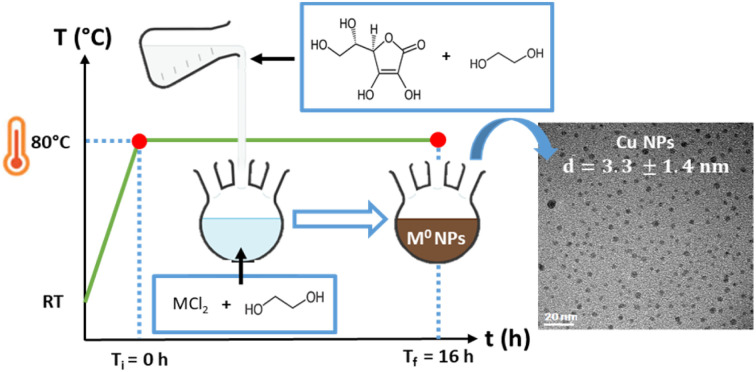

## 1. Introduction

Non-noble metals are attracting growing attention as potential candidates for replacing the scarce and expensive precious platinum group metals (PGMs) that are already in excessive use for various industrial applications. Naturally abundant and relatively nontoxic and inexpensive metals, particularly copper and tin, offer some enhanced physicochemical properties at the nanoscale compared to their bulk metals. Thus, they may be considered as promising alternatives with potential for high impact on future sustainable processes in catalysis, energy storage, optics, electronics, photovoltaics, and magnetism.^1–6^

Unfortunately, this objective remains difficult to achieve due to the challenges in accessing oxidation-/corrosion-resistant zerovalent copper or tin nanoparticles in a small size range, uniform size distribution, and sufficient yields.^7^ So far, robust synthetic procedures for efficient nano-engineering of copper and tin are very few compared with the extensive and already established studies found on gold or silver.^8,9^

The “wet chemical reduction” route, classified among the bottom-up strategies, offers several advantages, such as tunable nanoparticle size and morphology, moderate cost, and suitability, for up-scaling, which are crucial for large-scale practical use.^10^ The size control depends on the nucleation and growth rates initiated by the reduction of metal ions in the liquid phase.^11^ Initially, the synthesis processes were developed on the basis of the studies conducted on noble metals. For instance, the well-known classic polyol process proposed by Fievet in the 1980s^12^ was successfully extended to the synthesis of various transition metal nanoparticles with controlled sizes.^13^ The reduction by polyols, such as ethylene glycol (EG) and diethylene glycol (DEG), which also act as capping agents, depends on the nature of the polyol, pH, and particularly the reaction temperature, which is typically high (>160 °C). For example, copper nanocrystals with an average diameter of 2 nm were obtained by Kawasaki *et al.*^14^ following a surfactant-free polyol method using ethylene glycol as the reducing agent, which was activated by microwave radiation at a high temperature of 185 °C in the presence of NaOH. Further examples include the use of strong reducing agents, such as hydrazine, sodium borohydride and sodium hypophosphite, that are efficient at low temperatures.^15,16^ Nevertheless, concerns have quickly raised regarding the presence of impurities, chemical instability and potential hazard to the human health and environment.^17^

In addition, special efforts are needed to ensure the chemical stability of the produced nanoparticles (NPs) against oxidation by the air or water during synthesis, storage or processing steps. For instance, it was observed that Cu NPs synthesized by the thermal decomposition of copper acetylacetonate in oleylamine at 230 °C under an N_2_ atmosphere were quickly coated with a cuprous oxide (Cu_2_O) layer upon exposure to the air.^18^ Sn NPs are also naturally coated with an amorphous layer of SnO_2_.^19^ To remedy this issue, typical, heavy surface-protective agents, such as polyvinylpyrrolidone (PVP) and long-chain amines, are employed, but they are then difficult to remove.^20–23^ This highlights the urgent need for optimal synthetic conditions to produce pure metallic nanoparticles that are inherently resistant to oxidation and corrosion, without relying on heavy surface-capping agents.

In alignment with the principles of green chemistry, biocompatible, biodegradable, and non-toxic reducing agents, such as plant, vegetable, and fruit extracts, are increasingly emerging as promising green formulations that can be used under mild conditions.^24^ Ascorbic acid (AA, C_6_H_8_O_6_), an antioxidant extracted from fruits and vegetables, acts as an efficient reducing agent in the synthesis of metal nanoparticles.^25^ It is a mild reducing agent, and its reactivity depends strongly on the pH (*E*(C_6_H_8_O_6_) = 0.46 − 0.07pH (V/SHE) at 1 ≤ pH ≤ 13).^17,26^ The oxidation of AA leads to the formation of dehydroascorbic acid (DHA) *via* a 2-electron pathway. Since AA is a diprotic acid (H_2_A), depending on the pH, it can undergo two deprotonation steps to first form an ascorbate ion HA^−^ (p*K*_a_ = 4.2) and then an ascorbate dianion A^2−^ (p*K*_a_ = 11.8). Consequently, the redox potential of the DHA/AA couple strongly decreases with an increase in pH, and AA is thus considered a weak reducing agent in non-alkaline solutions.^27^ Thus, the pH of the reaction medium certainly has an effect on the reduction rate, and consequently, the subsequent nucleation and growth process is faster in basic media than in acidic media.^17^ Obviously, reactions conducted in a non-alkaline medium require high temperatures to accelerate the kinetics of the redox reaction and decrease the redox potential of ascorbic acid. Finally, the redox reaction depends also on the concentration of O_2_ and the nature of the metal species in the medium.^28^ AA is, in general, used as a reducing agent in the presence of stabilizers, such as citrate, sodium dodecyl sulphate and polyvinylpyrrolidone, in aqueous solutions.^29,30^ However, it can act as both a reducing agent and a capping agent. Xiong *et al.*^31^ proposed that the polyhydroxyl structure of DHA, resulting from the oxidation of AA followed by an hydrolysis in water, facilitates the complexation of copper nanoparticles and their stabilization by forming hydrogen bonds. They demonstrated that it is possible to obtain a highly stable dispersion of copper nanoparticles with a narrow size distribution (*d* < 2 nm) by reducing copper chloride in water using l-ascorbic acid at 80 °C.^31^ The obtained nanoparticles were characterized by UV-visible spectroscopy, Fourier transform infrared (FTIR) spectroscopy, and transmission electron microscopy (TEM). To the best of our knowledge, while the colloidal stability has been proven previously, the oxidation state of the nanoparticles has not yet been characterized.

In this work, we report a novel synthesis strategy for the preparation of sub-10 nm tin nanoparticles in ethylene glycol using ascorbic acid as the sole reducing agent, starting from a classical precursor salt and without any surfactant. By optimizing the solvent conditions initially developed for the synthesis of stable zero-valent Cu nanoparticles, we successfully extended the method to tin, obtaining highly stable suspensions of sub-10 nm particles with remarkable oxidation resistance. Notably, the unconventional α-Sn phase was formed instead of the commonly observed β-Sn, highlighting both mechanistic and material novelty. The obtained nanoparticles may be used in various fields, including printed electronics, conductive inks, battery materials, catalysis and optoelectronics, since α-Sn is a semiconductor-like material similar to SnO_2_.

## 2. Experimental

### 2.1. Materials

Copper(ii) chloride dihydrate (CuCl_2_·2H_2_O, 99%, Alfa Aesar), tin chloride dihydrate (SnCl_2_·2H_2_O, 99%, Sigma), l-ascorbic acid (C_6_H_8_O_6_, ≥99%, Sigma-Aldrich), ethylene glycol (C_2_H_6_O_2_, 99%, Sigma), diethylene glycol (C_4_H_10_O_3_, ≥99%, Roth) and hydrochloric acid (HCl, 35%, VWR) were purchased and used as received without further purification. The water used in the experiments was ultrapure (resistivity ≥ 18.2 MΩ cm).

### 2.2. Synthesis protocol

#### 2.2.1. Synthesis of copper nanoparticles

The general procedure involved the preparation of a 4 mmol solution of the copper precursor by vigorously dissolving it in 20 mL of a selected solvent (water, ethylene glycol (EG) or diethylene glycol (DEG)). The solution was loaded into a round-bottom flask equipped with a bubbler and was heated up to 80 °C in an oil bath under continuous stirring. When the temperature stabilized at 80 °C, a well-dissolved solution of 8 mmol l-ascorbic acid (AA) in 20 mL of the same solvent was quickly added to the pre-heated copper precursor solution. The concentration of Cu^2+^ in the solution was 0.1 mol L^−1^, and the molar ratio of AA/Cu^2+^ was 2. The reaction was then allowed to proceed for 16 h. A schematic describing the aforementioned procedure is presented in Fig. S1.

Following this protocol, several reactive mixtures were tested, with the solvent being the only experimental parameter that was varied. Each reaction mixture was subjected to a 16 h reaction at 80 °C. It is worth noting that, in some experiments, a specified volume of 35% HCl was added.

#### 2.2.2. Synthesis of tin nanoparticles

The same procedure described above for the synthesis of Cu NPs was applied to the synthesis of Sn NPs. Tin chloride dihydrate (SnCl_2_·2H_2_O), l-ascorbic acid and ethylene glycol were used as the precursor salt of tin, the reducing agent and the solvent, respectively. The molar ratio between l-ascorbic acid and tin(ii) ions was maintained at 2, as mentioned above.

### 2.3. Characterization

UV-visible spectra were recorded in a wavelength range of 200–900 nm on a Varian-Agilent Carry 5000 UV-NIR spectrophotometer.

Standard transmission electron microscopy (TEM) analysis was performed on a JEOL 2100 operated at 200 kV to check the NP formation after synthesis and to establish the particle diameter histograms. The aqueous suspensions were directly loaded onto a holey carbon film of Au grids (300 mesh) and then dried, while the suspensions prepared in polyalcohols were first diluted in ethanol at a 1 : 1 volume ratio. A minimum of 200 particles were counted to establish representative size histograms and determine the mean particle diameter.

High-resolution TEM/STEM imaging for the characterization of single NPs with atomic resolution was performed using a JEOL ARM 200F double aberration-corrected microscope operated at 200 kV. For high-resolution imaging, the colloidal suspensions of Cu and Sn NPs in ethylene glycol were first diluted in ethanol at a 1 : 4 ethylene glycol-to-ethanol ratio. For TEM/STEM observations, 2 μL of the diluted suspensions was drop-casted on nickel (for Cu NPs) or copper (for Sn NPs) holey-carbon TEM grids.

X-ray photoelectron spectroscopy (XPS) measurements were recorded using a Kratos Axis Ultra DLD equipment with a monochromatic Al X-ray source (1486.6 eV, 10 mA, 15 kV). To collect enough signal, droplets of colloidal suspensions were deposited either directly on a steel XPS sample holder (Cu NPs in EG) or on a TEM Au grid, which was then attached to a sample holder using an Ag paste (Sn NPs in EG). The samples were dried before being placed in the apparatus. The spectra were obtained using a pass energy of 20 eV over an analysis area of 300 μm × 700 μm. The spectral region corresponding to the Cu 2p or Sn 3d core levels was recorded for each sample. Data were acquired at 0.1 eV steps. Data processing was performed using the CasaXPS software.

Attenuated total reflection Fourier transform infrared spectroscopy (ATR-FTIR) was performed using a Thermo Nicolet 5700 spectrometer. The samples were analysed directly without further preparation; a droplet of each suspension was simply poured on a diamond disk and pressed with a steel probe for analysis. The analysis was performed in the spectral range of 650–4000 cm^−1^ with a spectral resolution of 2 cm^−1^. A background spectrum was first recorded, followed by the sample spectrum, which was averaged over 64 successive scans.

Inductively coupled plasma optical emission spectroscopy (ICP-OES) was used for the determination of metal concentration in the aqueous and organic metal suspensions. The liquid samples did not require acid digestion and were only diluted using ultrapure water. The analysis was performed on a 5110 Agilent VDV spectrometer.

## 3. Results and discussion

### 3.1. Synthesis of copper nanoparticles (Cu NPs)

#### 3.1.1. Reduction of Cu(ii) precursor in aqueous medium

Amid growing enthusiasm for developing green and eco-friendly methods for synthesizing monodisperse metal nano-colloids, based on the work of Xiong *et al.*,^31^ we initially selected water as the reaction solvent, as it is naturally abundant and dissolves a wide range of common metal precursors. Water also dissolves O_2_ from the air, which presents a challenge in yielding oxidation-resistant metal particles. One can note that the experiments were carried out in a closed flask (Fig. S1).

**Fig. 1:**
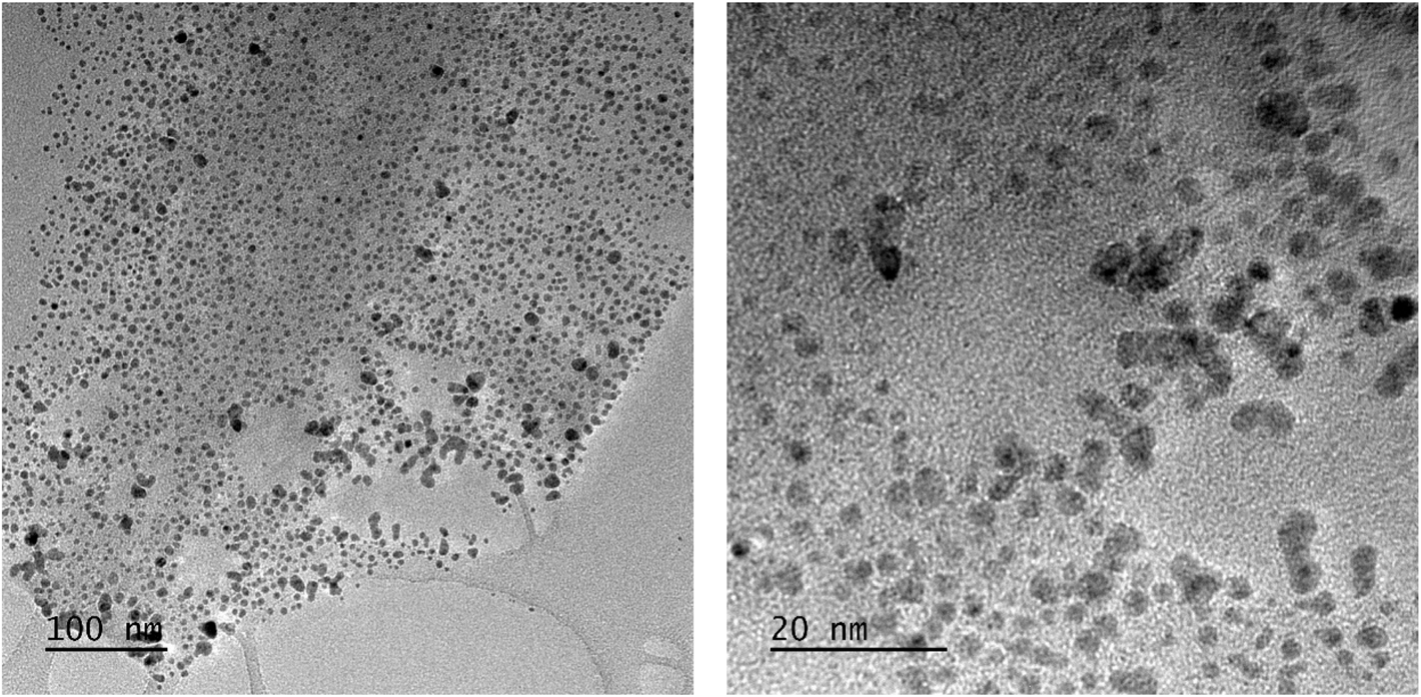
TEM images of Cu NPs obtained from the reduction of CuCl_2_ by AA in water at 80 °C for 16 h.

When the solution of AA was added to the solution of CuCl_2_ precursor, the light turquoise aqueous solution (pH = 2) instantly turned transparent, and a white solid was formed simultaneously. This result suggests a very fast partial reduction of Cu(ii), which was initially present as a hexaaqua copper complex [Cu(H_2_O)_6_]^2+^, to Cu(i), following the introduction of l-ascorbic acid. The obtained white solid contained around 74% of Cu by molar content, which was deduced from the quantification of the residual Cu in the liquid supernatant by ICP-OES, and it was attributed to the cuprous chloride (CuCl) powder.^32^ The appearance of this white solid was not mentioned by Xiong *et al.*^31^ A higher AA/[Cu(ii)] ratio of 6, instead of 2, was tested, but the formation of CuCl could not be avoided. Normally, Cu(i) ions are unstable in water; they are easily converted into Cu(ii) and Cu(0) or reduced to Cu(0) in the presence of a reducing agent. However, the reduction of the CuCl intermediate is difficult due to its very low solubility constant in water. During the reaction, the CuCl solid did not disappear, while the transparent supernatant changed to yellow, orange and finally brown after 16 h of reaction (Fig. S2). The TEM images of the brown supernatant are presented in Fig. 1, showing Cu NPs with sizes ranging from 1 to 15 nm.

In order to suppress the formation of the white solid of CuCl, a complementary experiment was performed by adding 5 mL HCl (35%) to the CuCl_2_ aqueous solution in order to have an excess of Cl^−^ in the medium. The pH of the reaction medium shifted from 2 to 1. Thus, even though it has been demonstrated that in the presence of chloride ions in the medium, the reduction of Cu^2+^ favours the one-electron redox process stabilizing the Cu(i) oxidation state,^33^ the formation of the white CuCl solid was not observed upon the introduction of AA due to the formation of soluble Cu(i) chloro-complexes, namely, CuCl_2_^−^, CuCl_3_^2−^ and CuCl_4_^3−^, in the presence of excess Cl^−^.^34,35^ However, only large Cu NPs with a mean diameter of 26.8 ± 7.9 nm were observed in TEM images (Fig. S3). In addition, the colloidal suspension was unstable, with the nanoparticles gradually coalescing to form a surface coating at the bottom of the reaction flask within a few days.

In conclusion, water was not the most suitable solvent for the synthesis of sub-10 nm Cu NPs, especially when ascorbic acid was used as the reducing agent.

#### 3.1.2. Reduction of Cu(ii) precursor in organic medium

**Fig. 2:**
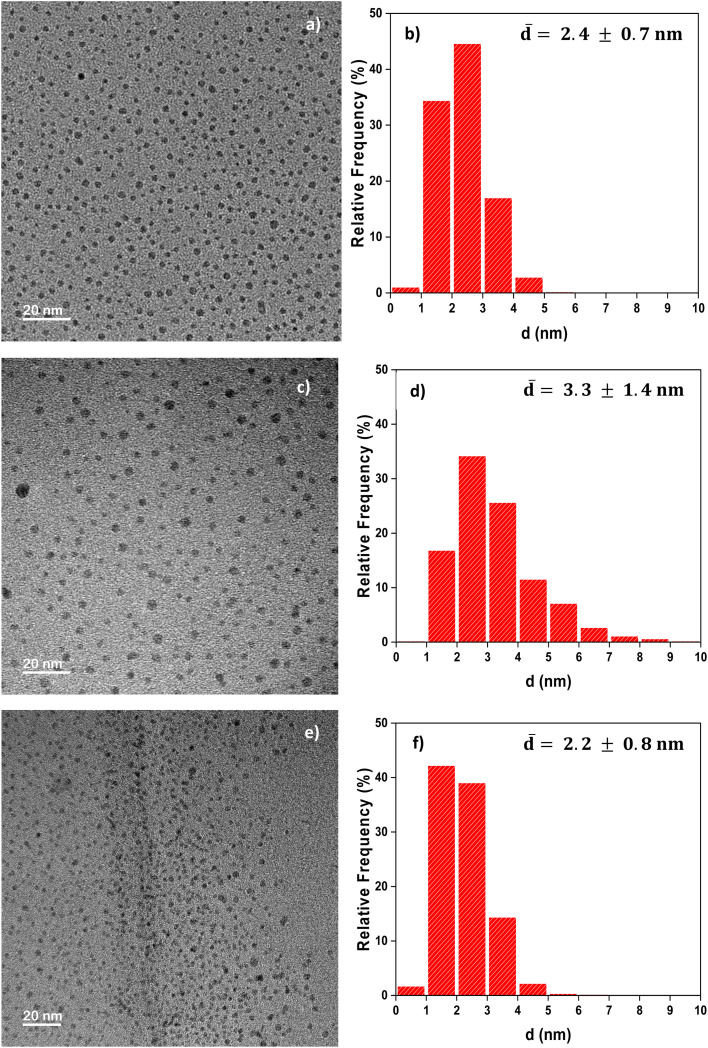
TEM images and particle diameter histograms of Cu NPs obtained from the reduction of CuCl_2_ by AA in various solvents at 80 °C for 16 h: (a and b) ethylene glycol, (c and d) ethylene glycol with the addition of 0.4 mL of HCl (35%) and (e and f) diethylene glycol.

Ethylene glycol (EG) was chosen as the organic solvent since the CuCl_2_ precursor salt dissolves well in it. EG is able to reduce metal ions at high temperatures, but below 120 °C, it presents a limited reducing power. Overall, in contrast with water, EG is a versatile solvent and can play the role of a bidentate ligand, forming complexes *in situ* during reactions, in addition to the role of a surface agent because of the non-bonding doublets of its alcoholic (–OH) functional group.^36^ In this solvent, the predominant Cu(ii) species is the [CuCl_2_(EG)_2_] complex, which is characterized by an intense green color.^37^ This complex is very stable and non-reducible even at a temperature as high as 210 °C, higher than the boiling point of EG (197.6 °C), which is usually used to enhance the reducing effect of EG.^38^ When AA was added to the solution of CuCl_2_ in EG at 80 °C, the uniform green solution underwent changes analogous to those observed in the aqueous medium, with CuCl solid as the insoluble intermediate (Fig. S4), indicating that the [CuCl_2_(EG)_2_] complex is likely to be destabilized when the reduction reaction occurs. In contrast to water, the brown supernatant was darker and composed of monodisperse Cu NPs with an average size of 2.4 ± 0.7 nm, as seen in the TEM image and size histogram presented in Fig. 2(a) and (b). The darker brown shade in EG is a qualitative indication of a higher Cu concentration in the supernatant. According to the ICP-OES analysis, the sedimented CuCl represented only 19% of the total Cu concentration in the reactive mixture. When 0.4 mL of HCl (35%) was added to the precursor solution before reaction, the outcome of the synthesis improved due to the excess of Cl^−^ ions, as observed in water (Fig. S4). Note that in ethylene glycol, a smaller amount of Cl^−^ (0.4 mL instead of the previously used 5 mL HCl in water) was sufficient to prevent the formation of insoluble CuCl. The resulting Cu NPs retained perfect homogeneity, with a slight increase in the average diameter (3.3 ± 1.4 nm) compared to those obtained in the absence of HCl, as shown in Fig. 2(c) and (d). This is due to the greater amount of Cu^2+^ reduced, which promotes nanoparticle growth. Notably, the suspensions of copper NPs thus obtained were very stable over time, with no destabilisation after 12 months of storage.

**Fig. 3:**
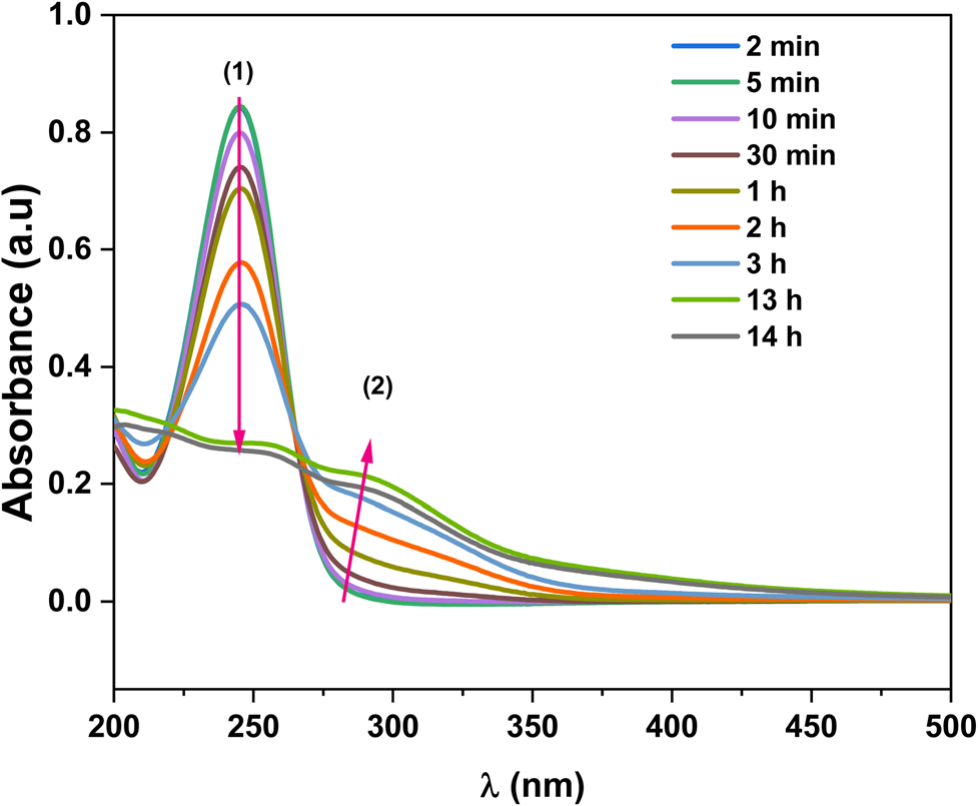
UV-visible spectra representing the evolution of the CuCl_2_ reduction by AA with time at 80 °C in ethylene glycol in the presence of 0.4 mL HCl (35%).

To monitor the progress of the reaction over time, aliquots of the Cu NPs suspensions prepared in EG with added HCl were withdrawn at different times of the reaction and analyzed by UV-visible spectroscopy after dilution (Fig. 3). The spectra were compared to those of fresh solutions of AA dissolved in EG and CuCl_2_ dissolved in EG (Fig. S5). The fresh solution of AA in EG displayed an absorption peak at *ca.* 245 nm (indexed as (1) in Fig. 3). The recording of absorption spectra of the reaction mixture started at 2 minutes of the reaction, slightly after the addition of AA to the Cu(ii) solution. The difference in absorbance between the 2 minutes spectrum and the fresh AA can be associated with the reduction of Cu^2+^ to Cu^0^. Then, for the reactive mixture of the Cu NPs suspension, the peak representative of AA in EG continuously decreased during the reaction (downward pink arrow for peak (1)), indicating slow and gradual degradation of ascorbic acid due to the slow reduction of Cu^2+^ or Cu^+^. After 13 hours of the reaction, peak (1) completely disappeared, suggesting that the reaction was likely complete due to the total consumption of AA from the medium. In addition, the spectrum stabilization between 13 h and 14 h suggests that the Cu NP nucleation/growth had reached equilibrium at 13 h. Notably, the resulting spectrum attributed to the Cu NPs formed was characterized by the gradual appearance of a band at *ca.* 284 nm (upward pink arrow at the peak labelled (2)). The absorption of the presumably formed Cu NPs did not show similarities with that recorded for the starting Cu^2+^ ions.

In the experimental conditions chosen, especially at reaction temperatures below 100 °C, the oxidation of EG is unlikely. This was checked by infrared spectroscopy analysis of the suspension after 16 h of reaction in the presence of HCl. The ATR-FTIR spectrum (Fig. S6) of the Cu NPs suspension displayed the same absorption bands and the same intensities as the spectrum of fresh EG (grey line), and the well-known EG oxidation products (acetaldehyde, glycolaldehyde, and glyoxal) were not detected.^13^ Consequently, it can be inferred from the UV-Visible and ATR-FTIR spectra that the reducing agent of Cu^2+^ was likely AA, and not EG, as reduction also occurred in water.

**Fig. 4:**
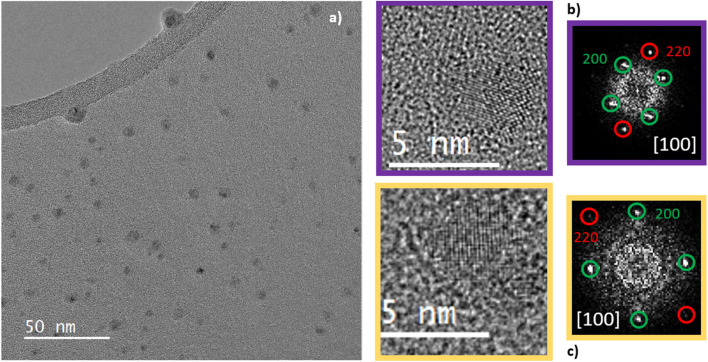
(a) HRTEM image of the Cu NPs synthesized in EG and 0.4 mL HCl (35%) after H_2_ treatment at 300 °C for 1 hour. (b and c) HRTEM images of two representative Cu NPs in the [001] zone axis with their indexed FFT.

The direct access to crystallographic information of the Cu NPs dispersed in organic solvent using HR TEM or HR STEM-HAADF was limited due to the organic contamination under the electron beam, which acted as a veil, as shown in Fig. S7. Consequently, to remove the organic solvent, the TEM grids used for high-resolution TEM/STEM imaging were heated under H_2_ at 300 °C for 1 h (temperature ramp 10 °C min^−1^) prior to observation. Despite this cleaning procedure, organic contamination during TEM analysis could not be entirely eliminated, and thus, electron microscopy imaging of the Cu NPs was performed in the TEM mode to minimize contamination of the sample under the electron beam (Fig. 4(a)). Fig. 4(b) and (c) show the high-resolution TEM images of two representative NPs. The Cu NPs have an average size of 4 nm, possibly due to interparticle sintering at 300 °C. The corresponding fast Fourier transform (FFT) is shown in the inset. The interplanar distances and interplanar angles obtained from the TEM images were consistent with the face-centred cubic (FCC) structure of copper, as viewed along the [001] zone axis. For instance, the interplanar distances obtained from the analysis of the FFT image of the NP in Fig. 4(b) (1.28 Å for (220) planes (red) and 1.84 Å for (200) planes (green)), as well as the interplanar angles between these planes (45° between the (220) and (200) planes), match well with those of the FCC phase of bulk Cu (1.27 Å for (220) planes and 1.80 Å for (200) planes). This structural analysis also rules out the presence of a crystallized oxide copper phase in the sample.

In order to confirm that the synthesis of Cu NPs in EG using AA as the reducing agent yields zerovalent copper metal (Cu^0^), the suspension was further analysed by XPS (Fig. S8(a)). The peaks of Cu 2p_3/2_ and Cu 2p_1/2_ were located at 932.2 eV and 952.1 eV, respectively, showing that Cu(ii) (at *ca.* 933.8 and 953.8 eV, respectively) was not present. This confirms that Cu was present in a reduced state, likely as Cu(0), in agreement with the TEM results. However, the presence of Cu(i) cannot be entirely ruled out, as its binding energies differ only by approximately 0.1 eV compared with those of the Cu(0) species.^39^

**Fig. 5:**
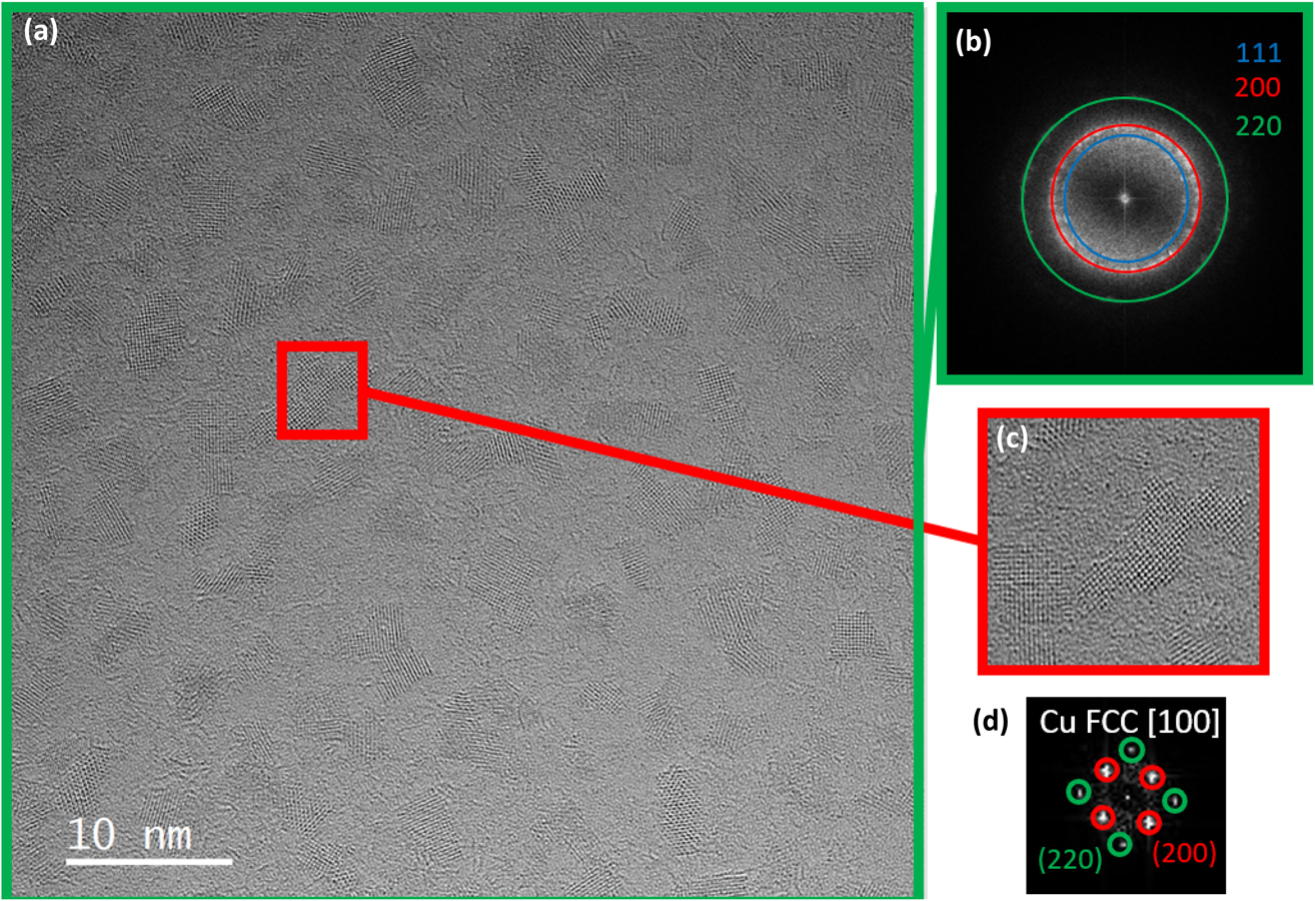
(a) HRTEM image of Cu NPs synthesized in DEG. (b) Reduced Fourier transform of the whole area showing the three families of planes with lower indices for FCC structures: (200) (red), (111) (blue) and (220) (green). (c) HRTEM image of a specific area of (a) containing a NP. (d) Reduced Fourier transform of the NP in (b) matching with FCC Cu in the [100] direction.

Diethylene glycol (DEG), a four-carbon dimer of EG, dissolves the CuCl_2_ salt as well and presents a higher chelating property than EG due to the ether functional group (–O–). Consequently, it was also tested as a solvent and chelating agent in the synthesis of Cu NPs. The reduction of CuCl_2_ by AA in diethylene glycol at 80 °C resulted in a uniform brown solution (Fig. S4). Interestingly, the common intermediate CuCl solid observed so far was visibly not formed, and the excess Cl^−^ ions from added HCl were not required anymore. The resulting uniform brown solution in DEG corresponds to highly stable, small-sized Cu nanocrystals measuring around 2.2 ± 0.8 nm, as highlighted in Fig. 2(e) and (f). As seen in Fig. 5, these nanoparticles were flattened; their thickness was lower than that of the Cu NPs synthesized in the EG medium. Their plate-like shape is due to the modified adsorption characteristics of DEG, which has 2 types of “O” moieties: alcohol and ether.^37^ Chemically, the NPs corresponded to the FCC structure of Cu, as highlighted by the diffraction spots of the (200) and (220) planes in the HRTEM image.

Overall, the Cu NPs synthesized in either of the diols (EG and DEG) displayed fascinating colloidal stability for more than 12 months and could not be destabilized by centrifugation even at higher rotation speeds (11 000 rpm) and longer duration (1 h). The measurement of *ζ*-potential helps in understanding the mechanism of the colloidal stability; however, dilution with water was necessary to reach a satisfactory electrical conduction for analysis in solutions containing non-electrolytic organic solvents. The water-diluted suspension of Cu NPs synthesized in EG remained stable and retained the uniform brown colour, and the *ζ*-potential value was 0 V. This result demonstrates the steric stabilization of the Cu NPs due to the effect of EG molecules, potentially along with that of dehydroascorbic acid, indicating that the negatively charged Cl^−^ ions present in the medium do not play a role. In contrast, the suspension of Cu NPs synthesized in DEG immediately turned green upon water addition, and a white solid was formed, suggesting the reversibility of the NP formation process, in a fashion comparable to that of a purely aqueous medium, as discussed earlier. Thus, these NPs are highly sensitive to oxidation by water, leading to further corrosion/dissolution. DEG at the surface of the NPs is permeable to O_2_ diffusion, and this explains the harsh corrosion phenomenon.^34^ Consequently, among the solvents tested, only EG can prevent Cu NPs from oxidation.

**Fig. 6:**
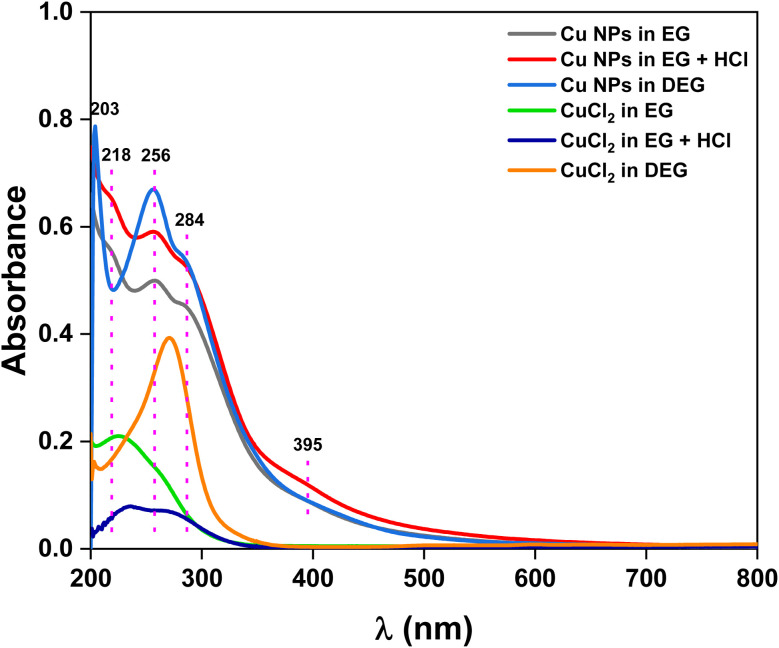
UV-visible absorption spectra of the as prepared Cu NPs in different solvent conditions.

Finally, to verify the formation of sub-10 nm Cu NPs in the polyol media (EG, EG with HCl, and DEG), UV-visible absorption spectra were recorded at the end of the experiment, *i.e.*, after 16 h of the reaction (Fig. 6). First, the absence of a localized surface plasmon resonance (LSPR) peak in the 550–650 nm spectral range in the three media was proof of the absence of large copper nanoparticles.^40^ In addition, the absorption bands highlighted with the dashed pink line at 218, 256, 284 and 395 nm (Cu NPs in EG), and those at 203, 256 and 284 nm (Cu NPs in DEG) were characteristic of small NPs.^14,40,41^ Besides, they do not correspond to the absorption bands of CuCl_2_ dissolved in the same solvent at the same concentration, in the absence of AA. The position and shape of these bands are influenced by the molecular environment at the metal surface and closely align with those reported by Kawasaki *et al.*, for Cu NPs with diameters less than 3 nm.^14^ Comparatively, when HCl was added to the synthesis medium to suppress CuCl precipitation and enhance the efficiency of Cu^2+^ reduction, the absorbance increased (from grey to red spectrum) because of the rising concentration of Cu nanocrystals in the solution.

### 3.2. Synthesis of tin nanoparticles (Sn NPs)

**Fig. 7:**
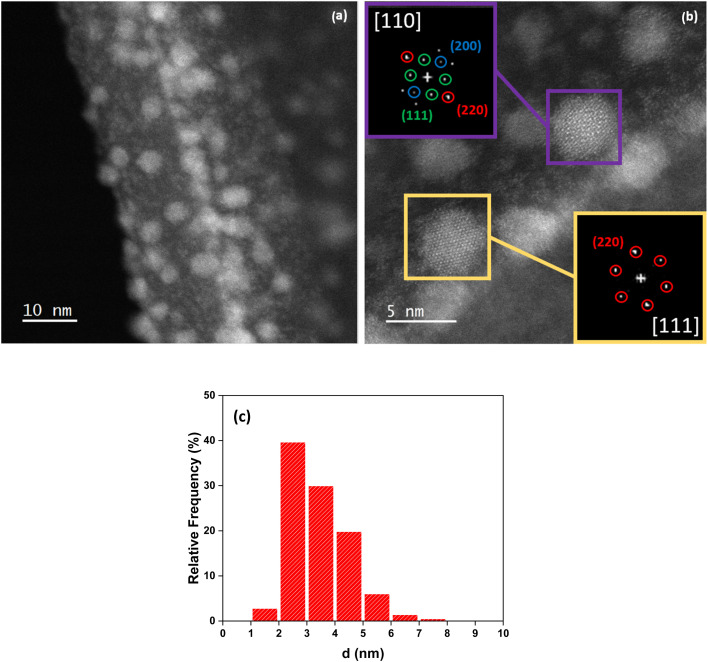
STEM-HAADF images of Sn NPs dispersed in ethylene glycol after the reaction of SnCl_2_ with AA at 80 °C for 16 h. (a) Low resolution and (b) high resolution images of two Sn NPs in [110] and [111] zone axes with their indexed FFT. (c) Particle diameter histogram.

Sn NPs obtained through various synthetic routes are subjected to systematic oxidation upon exposure to air. An oxidation shell, often made of stannic oxide (SnO_2_), is formed spontaneously.^42,43^ As EG has demonstrated to be an efficient solvent for obtaining small nanoparticles of Cu, the synthesis of Sn NPs was performed in the same solvent. SnCl_2_ dissolved easily in EG, resulting in a colourless solution. When AA was added to the colourless SnCl_2_ precursor solution, the reactive mixture gradually turned yellow, eventually displaying a uniform light golden-yellow colour after 16 h of reaction (Fig. S4). The Sn NPs were stable in the solvent and could not be precipitated by centrifugation. As shown in Fig. 7(a) and (c), small Sn NPs were obtained (average size ≈ 3.5 ± 1.2 nm). Fig. 7(a) and (b) show the high-resolution STEM-HAADF images of the Sn NPs. In Fig. 7(b), the atomically resolved FFT images of the two NPs are consistent with the α-Sn diamond-like structure. One is in the [110] zone axis (violet box), and the other one is oriented along the [111] zone axis (yellow box). Further, the interplanar distances measured (3.11 Å for (200) planes, 2.18 Å for (220) planes and 3.46 Å for (111) planes) are very close to those of α-Sn with lattice parameter *a* = 6.49 Å (interplanar spacings of 3.25 Å for (200) planes, 2.30 Å for (220) planes and 3.75 Å for (111) planes). Moreover, these FFT images are not consistent with the tetragonal structure of β-Sn, with *a* = 5.82 Å and *c* = 3.17 Å. The contours of the particles are poorly defined, probably due to the strong presence of adsorbed organic ligands. A few more particles, along with their structures, are presented in the SI (Fig. S9). XPS analysis also confirmed the presence of zerovalent Sn (Sn^0^), as well as tin oxide (SnO_2_), likely due to the formation of a passivation shell around the Sn^0^ NPs (Fig. S8(b)).

Interestingly, the Sn NPs resulting from colloidal synthesis reported in the literature crystallized into the white tin phase (β-Sn), which is a tetragonal system.^44,45^ The stabilization of Sn NPs in the grey tin phase (α-Sn), which displays a diamond (FCC) cubic lattice, has rarely been observed under conventional conditions of wet chemistry syntheses.^46,47^ The change in the crystalline system modifies the metal properties; β-Sn has metal-like properties, while α-Sn is likely a semiconductor. This unusual result may be synthesis-mediated, demonstrating the strength and versatility of ethylene glycol (EG) as both a synthesis medium and, more specifically, a surface agent.

## 4. Conclusion

In summary, a straightforward colloidal approach was developed for the synthesis of stable copper nanoparticles under ambient conditions by employing a modified polyol process at 80 °C. This approach was successfully extended to the synthesis of tin nanoparticles, making it a generic method for the synthesis of other non-noble metals that are highly prone to oxidation. Ethylene glycol served as the solvent, while l-ascorbic acid functioned as the reducing agent. This method yielded sub-10 nm nanoparticles, with Cu and Sn predominantly in their zerovalent states, and with an unconventional phase for Sn. The resulting nanoparticles exhibited remarkable long-term stability against both aggregation and oxidation.

## Conflicts of interest

They are no conflicts to declare.

## Supplementary Material

RA-015-D5RA04557F-s001

## Data Availability

Data will be made available on request. Supplementary information is available. See DOI: https://doi.org/10.1039/d5ra04557f.
