# Supplementary material for: Facile and green synthesis of monodisperse sub-10 nm copper and tin nanoparticles using l-ascorbic acid as the reducing agent
Source: RSC Adv. 2025 Oct 1;15(43):36405–13. doi: 10.1039/d5ra04557f (PMC12486232; doi:10.1039/d5ra04557f)
Supplement: RA-015-D5RA04557F-s001 [file RA-015-D5RA04557F-s001.pdf]

Supplementary Information (SI) for RSC Advances.
This journal is © The Royal Society of Chemistry 2025

Supplementary information

Facile and Green Synthesis of Monodisperse Sub-10 nm Copper and Tin Nanoparticles Using L-ascorbic Acid as a Reducing Agent

Abdennour Benabbas[1]*, Grégoire Breyton[2], Catherine Especel[1], Anthony Le Valant[1], Christian Ricolleau[2], Guillaume Wang[2], Tzonka Mineva[3], Jaysen Nelayah[2], Hazar Guesmi[3], Florence Epron[1]*

1. CNRS, Université de Poitiers, Institut de Chimie des Milieux et Matériaux de Poitiers (IC2MP), Poitiers, France

2. Laboratoire Matériaux et Phénomènes Quantiques, Université Paris Cité - CNRS, Paris, France

3. Institut Charles Gerhardt de Montpellier (ICGM), Université de Montpellier, CNRS, ENSCM, Montpellier, France

*florence.epron@univ-poitiers.fr, abdennour.benabbas@univ-poitiers.fr

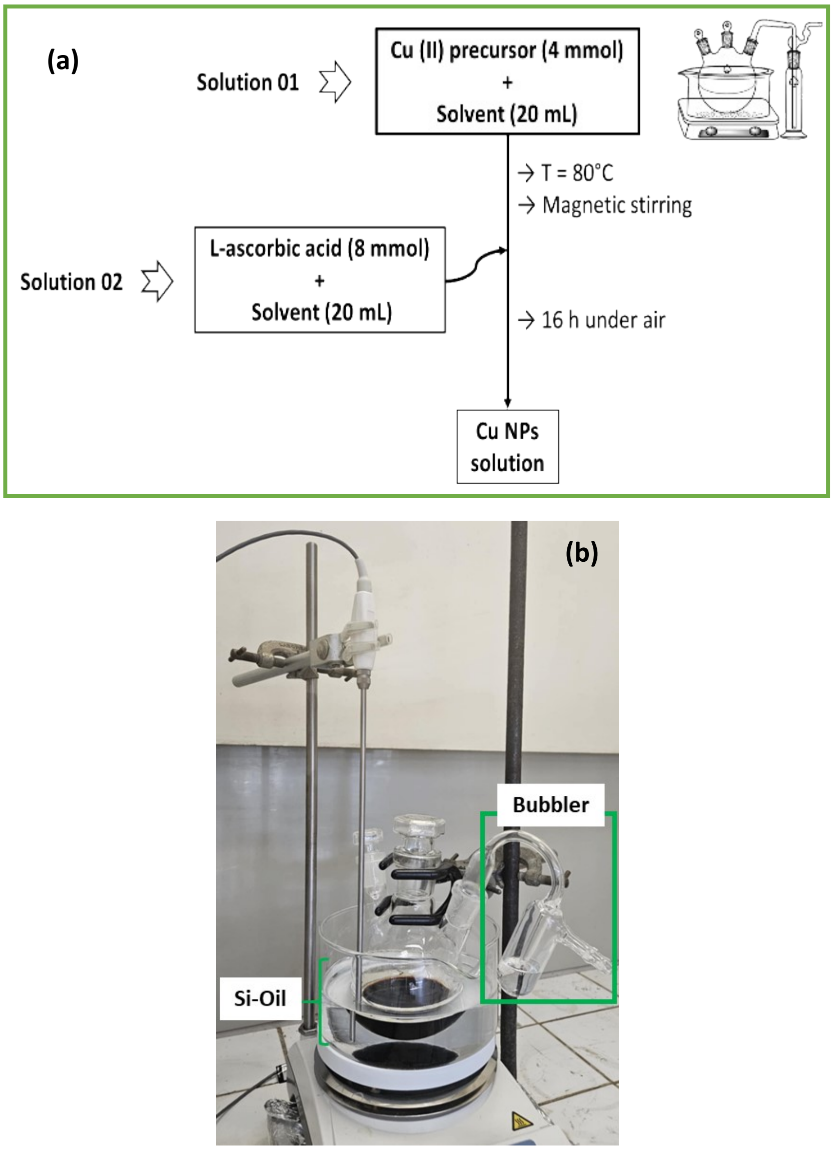

Figure S1. Schematic representation of the Cu NPs synthesis procedure (a) and Experimental set-up for the synthesis of Cu and Sn nanoparticles. The bubbler is filled with pristine EG. The heating bath is filled with Si-Oil. Example in the figure: Cu NPs synthesised in EG with HCl addition (35%) (b).

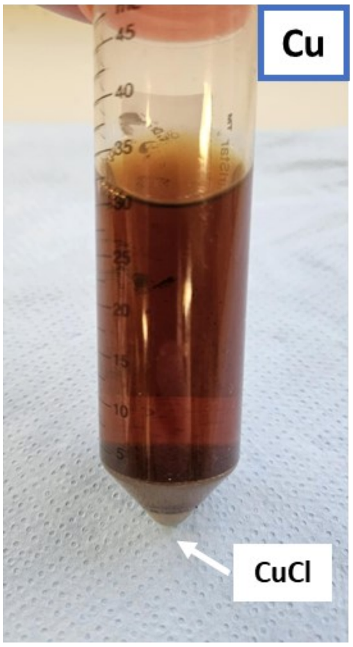

Figure S2. Representative images of the synthesis of Cu NPs after 16 h of reaction conducted in water.

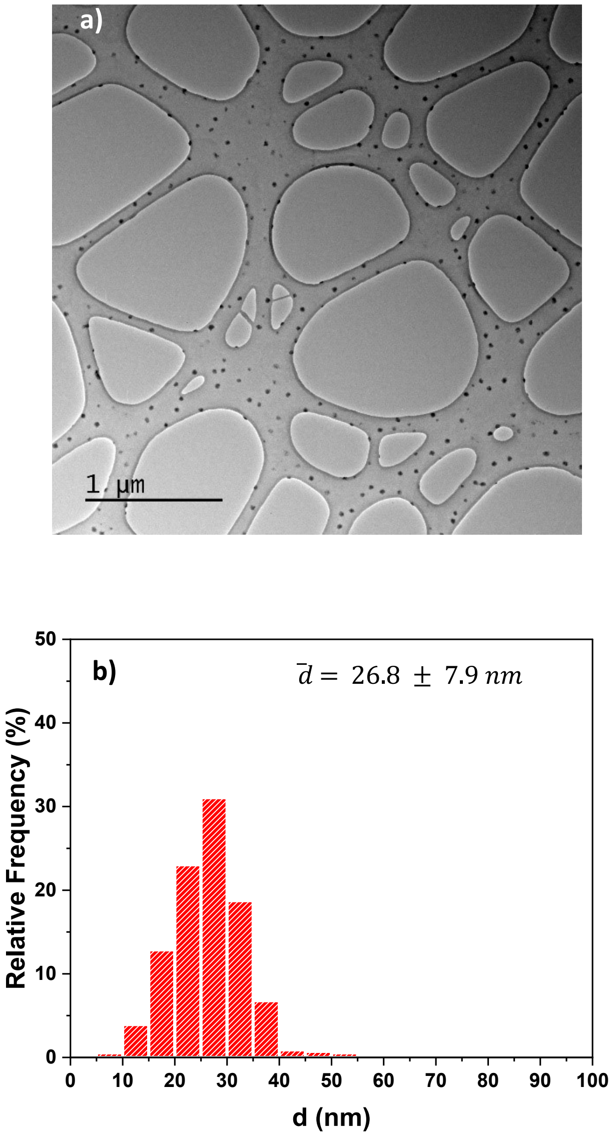

Figure S3. TEM image (a) and particle diameter histogram (b) of Cu NPs obtained from the reduction of $CuCl_2$ by AA in water with 5 mL of HCl (35%) at 80 °C for 16 h.

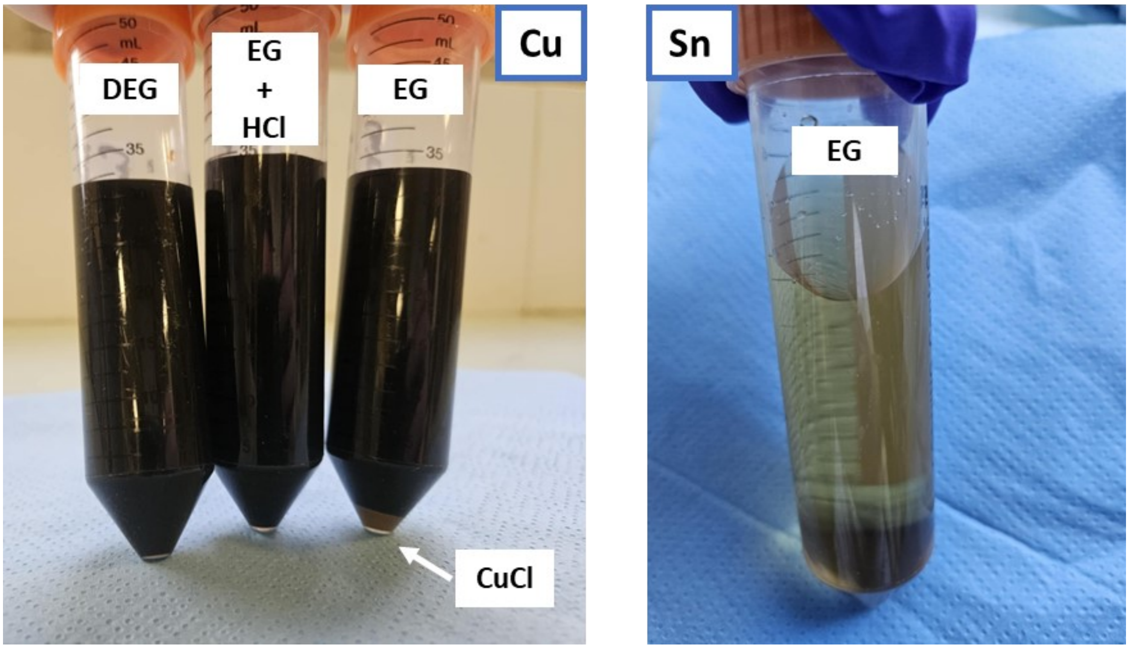

Figure S4. Representative images of the synthesis of Cu NPs (left) and Sn NPs (right) conducted in polyol media. Cu NPs were synthesised in: EG (with CuCl precipitate), EG + HCl, and DEG. Sn NPs were synthesised in EG.

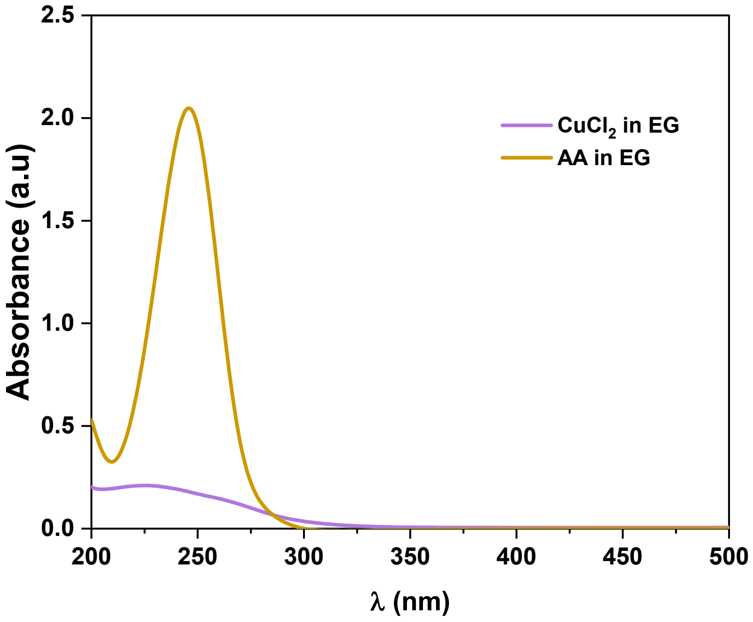

Figure S5. UV-visible spectra of $CuCl_2$ and ascorbic acid (AA) in ethylene glycol solution.

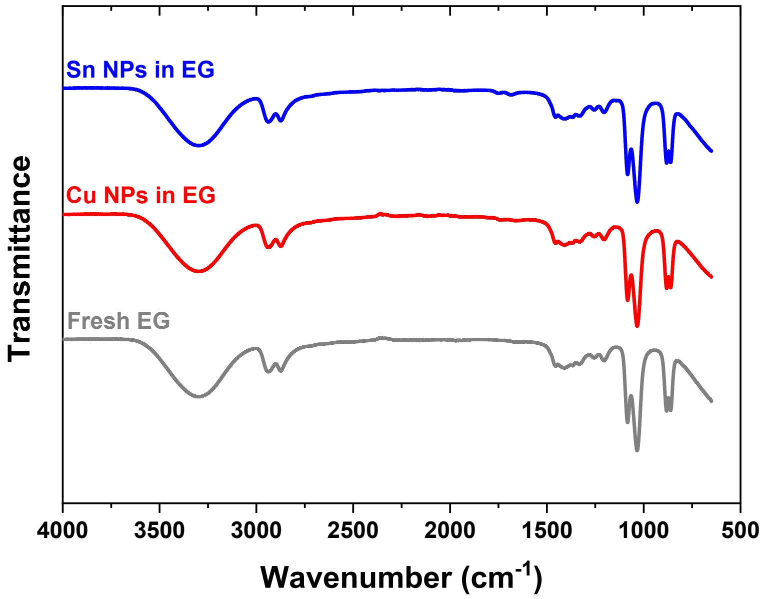

Figure S6. ATR-FTIR spectra of fresh ethylene glycol EG solvent (grey line), suspension of Cu NPs synthesized in ethylene glycol in the presence of HCl (red line) and suspension of Sn NPs synthesized in ethylene glycol (blue line).

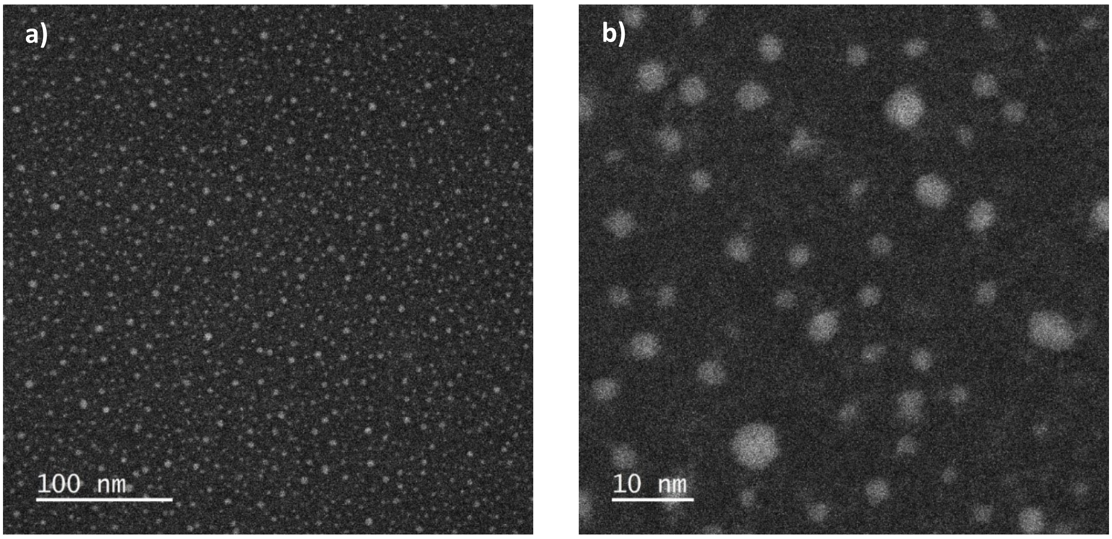

Figure S7. STEM-HAADF images of Cu NPs synthesized in ethylene glycol with the addition of HCl (0.4 mL) at low (a) and high (b) magnitudes.

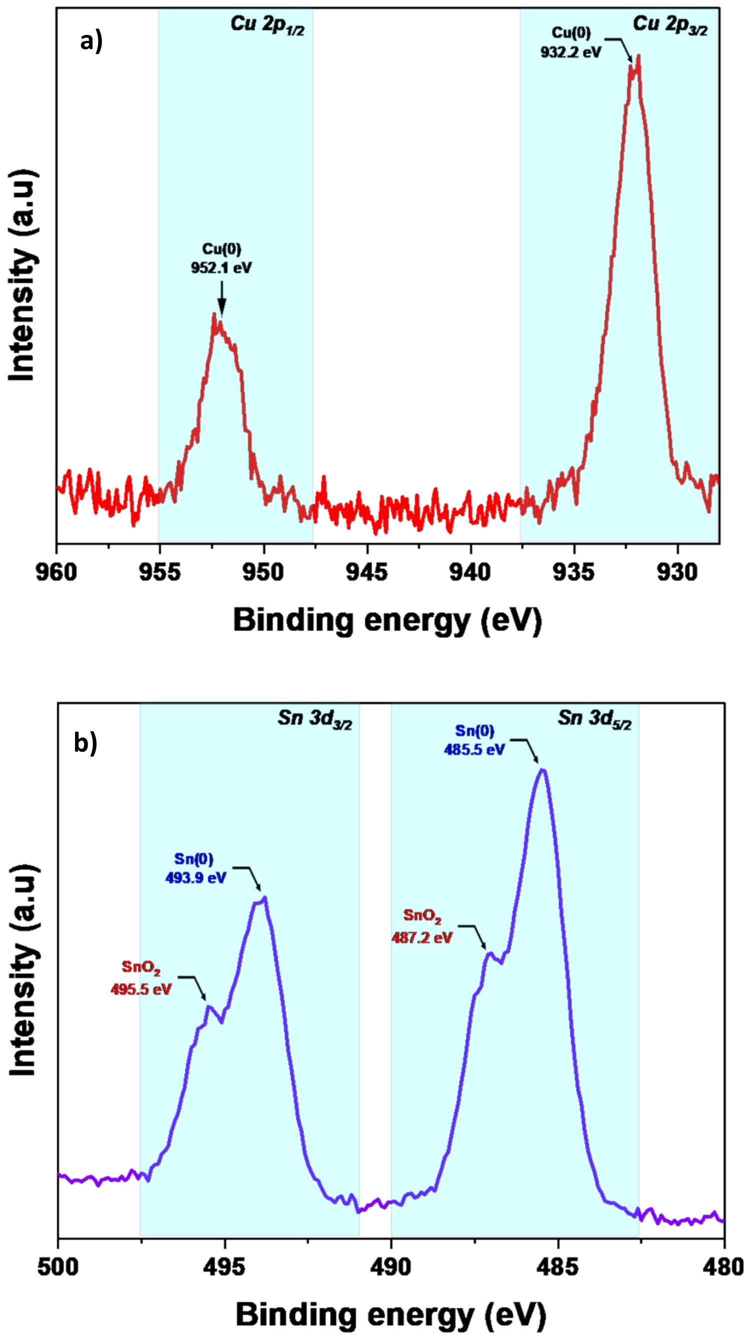

Figure S8. XPS spectra of: (a) Cu NPs (lines Cu $2p_{3/2}$ and Cu $2p_{1/2}$) synthesized in EG in the presence of AA and HCl and (b) Sn NPs (lines Sn $3d_{5/2}$ and Sn $3d_{3/2}$) synthesized in EG.

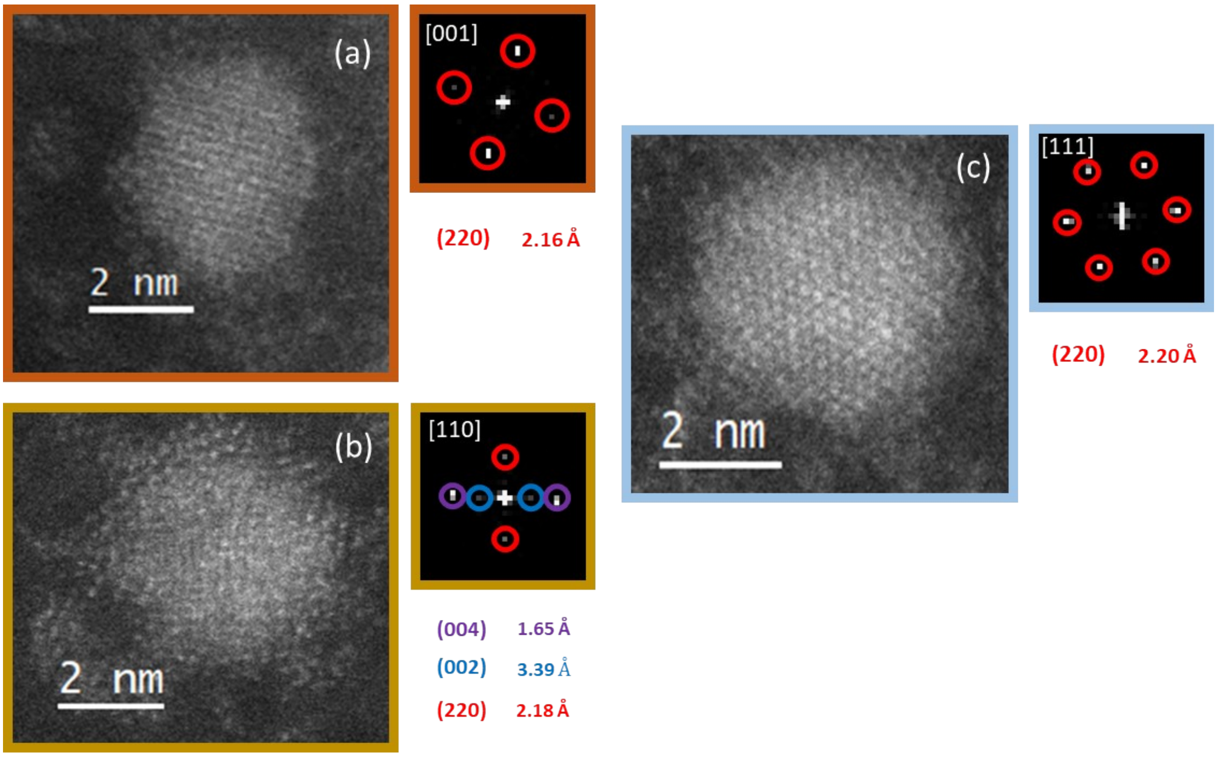

Figure S9. Supplementary STEM-HAADF images of Sn NPs dispersed in ethylene glycol after reaction with AA at 80 °C for 16 h. Sn NPs in figures (a), (b) and (c) are in [001], [110] and [111] zone axes respectively. The indexed FFT is given for each image according to a colour code.
